# Genomic Prediction of Kernel Zinc Concentration in Multiple Maize Populations Using Genotyping-by-Sequencing and Repeat Amplification Sequencing Markers

**DOI:** 10.3389/fpls.2020.00534

**Published:** 2020-05-08

**Authors:** Rui Guo, Thanda Dhliwayo, Edna K. Mageto, Natalia Palacios-Rojas, Michael Lee, Diansi Yu, Yanye Ruan, Ao Zhang, Felix San Vicente, Michael Olsen, Jose Crossa, Boddupalli M. Prasanna, Lijun Zhang, Xuecai Zhang

**Affiliations:** ^1^College of Agronomy, Shenyang Agricultural University, Shenyang, China; ^2^International Maize and Wheat Improvement Center (CIMMYT), Texcoco, Mexico; ^3^College of Biosciences and Biotechnology, Shenyang Agricultural University, Shenyang, China; ^4^Department of Agronomy, Iowa State University, Ames, IA, United States; ^5^CIMMYT-China Specialty Maize Research Center, Shanghai Academy of Agricultural Sciences, Shanghai, China; ^6^Crop Breeding and Cultivation Research Institute, Shanghai Academy of Agricultural Sciences, Shanghai, China; ^7^International Maize and Wheat Improvement Center (CIMMYT), Nairobi, Kenya

**Keywords:** maize, kernel Zn concentration, genomic selection, GBS, rAmpSeq

## Abstract

Enriching of kernel zinc (Zn) concentration in maize is one of the most effective ways to solve the problem of Zn deficiency in low and middle income countries where maize is the major staple food, and 17% of the global population is affected with Zn deficiency. Genomic selection (GS) has shown to be an effective approach to accelerate genetic gains in plant breeding. In the present study, an association-mapping panel and two maize double-haploid (DH) populations, both genotyped with genotyping-by-sequencing (GBS) and repeat amplification sequencing (rAmpSeq) markers, were used to estimate the genomic prediction accuracy of kernel Zn concentration in maize. Results showed that the prediction accuracy of two DH populations was higher than that of the association mapping population using the same set of markers. The prediction accuracy estimated with the GBS markers was significantly higher than that estimated with the rAmpSeq markers in the same population. The maximum prediction accuracy with minimum standard error was observed when half of the genotypes were included in the training set and 3,000 and 500 markers were used for prediction in the association mapping panel and the DH populations, respectively. Appropriate levels of minor allele frequency and missing rate should be considered and selected to achieve good prediction accuracy and reduce the computation burden by balancing the number of markers and marker quality. Training set development with broad phenotypic variation is possible to improve prediction accuracy. The transferability of the GS models across populations was assessed, the prediction accuracies in a few pairwise populations were above or close to 0.20, which indicates the prediction accuracies across years and populations have to be assessed in a larger breeding dataset with closer relationship between the training and prediction sets in further studies. GS outperformed MAS (marker-assisted-selection) on predicting the kernel Zn concentration in maize, the decision of a breeding strategy to implement GS individually or to implement MAS and GS stepwise for improving kernel Zn concentration in maize requires further research. Results of this study provide valuable information for understanding how to implement GS for improving kernel Zn concentration in maize.

## Introduction

Known as “hidden hunger,” micronutrient malnutrition is mainly prevalent among pregnant women and infants in the low and middle income countries (LMIC), where people rely mostly on cereal-based diets (Diepenbrock and Gore, 2015). Low levels of micronutrients, including zinc (Zn), iron and pro-vitamin A, lead to malnutrition-related health impairments (Cakmak, 2002; Tiwari et al., 2015). According to the World Health Organization, Zn deficiency affected 17% of the global population^1^. Zn micronutrient deficiency, prevalent among young children in developing countries, is associated with decreased immune-competence and increased rates of infectious diseases, which have been reported as an extensive food-related primary health problem in LMIC (Gibson, 1994; White and Broadley, 2009). Biofortification is a promising approach to improve micronutrient malnutrition through breeding and biotechnology, and enrich the micronutrient in the food by develop new varieties (Bouis and Saltzman, 2017).

The HarvestPlus project, a CGIAR research program, has been working to micronutrient malnutrition through bio-fortification of staple crops (Ortiz-Monasterio et al., 2007). Maize is one of the target crops of HarvestPlus, and the most important staple food for millions of people in major developing countries in sub-Saharan Africa, Latin America, and Asia (Jones and Thornton, 2003; Beyene et al., 2016). In maize, the baseline of kernel Zn concentration is about 20 mg/kg, the breeding target established by HarvestPlus project was 33 mg/kg, assuming the estimated average requirement of 1,860 μg per day of Zn in maize (Bouis and Welch, 2010). Therefore, an increase of at least 13 mg/kg is targeted by breeding. This target is achievable, due to the significant genetic variation for kernel Zn concentration exists in tropical maize germplasm, ranging from 4 to 96 mg/kg (Bänziger and Long, 2000; Ortiz-Monasterio et al., 2007; Prasanna et al., 2011, 2020; Hindu et al., 2018). Enriching the kernel Zn concentration in maize through bio-fortification is one of the most effective ways to solve the problem of Zn deficiency for pregnant women and young children living in the above-mentioned areas. Dissecting the genetic architecture of kernel Zn concentration in maize with genome-wide molecular markers will allow breeders to improve their breeding efficiency by facilitating the introgression of the related genes into low Zn germplasm through marker-assisted selection or genomic selection (GS). Several studies have been conducted to dissect the genetic architecture of kernel Zn concentration in maize (Qin et al., 2012; Simic et al., 2012; Baxter et al., 2013; Jin et al., 2013; Hindu et al., 2018). Jin et al. (2013) identified five significant QTL and ten meta-QTL in 218 *F*_2:3_ maize families. Hindu et al. (2018) detected 20 SNPs significantly associated with kernel Zn concentration in maize by implementing association mapping in a collection of 923 inbred lines, and 11 of those SNPs were validated in 3 DH populations by single marker linkage mapping analysis.

Genomic selection has been shown to be an effective approach to accelerate genetic gains in maize breeding (Lorenzana and Bernardo, 2009; Lian et al., 2014; Zhang et al., 2015, 2017; Cao et al., 2017). Highly variable prediction accuracy levels have been reported in plants depending on the training population size, the relationship between the training and the prediction sets, trait complexities, marker densities, and genotyping platforms (Zhao et al., 2012; Bian and Holland, 2017; Norman et al., 2018). Several studies have shown similar results, i.e., that prediction accuracy increases as trait heritability, size of training set, and number of markers in various types of maize populations increase (Heffner et al., 2009; Hickey et al., 2014; Liu et al., 2018; Norman et al., 2018). Norman et al. (2018) showed that prediction accuracy can be improved by broadening the genetic diversity within the training set, particularly when relatedness between training and validation sets is low. Genomic prediction analyses were conducted on a collection of 284 maize inbred lines, which were genotyped with both 1,148 and 55,000 SNPs. Results indicated that the prediction accuracies increased as the number of markers used across all the trait-environment combinations increased (Crossa et al., 2010; Gonzalez-Camacho et al., 2012).

An economical genotyping platform is always required in order to make GS more cost-effective. GBS, a next-generation sequencing technology, is a high-throughput, multiplex and short-read sequencing approach that reduces genome complexity via restriction enzymes and generates high-density genome-wide markers (∼1 million) at a low cost per sample by tagging randomly shared DNA fragments from different samples with unique, short DNA sequences (barcodes) and pooling samples into a single sequencing channel (Elshire et al., 2011; Wu et al., 2016). Several studies have indicated that GBS is a promising genotyping platform for GS applications (Poland et al., 2012; Crossa et al., 2013; Zhang et al., 2015; Yu et al., 2016). Zhang et al. (2015) showed that GBS outperformed low-density SNPs for both complex and simple traits evaluated under stress conditions with low-to-moderate heritability in 19 tropical maize bi-parental populations evaluated in multi-environment trials. Rio et al. (2019) obtained good prediction accuracies for grain moisture, grain yield, yield index and male flowering in a collection of 389 dent kernel type maize inbred lines that were genotyped with GBS and other sequencing technology. Developing a high-throughput genotyping platform with high quality, flexibility, and affordable genotyping cost is still critical for implementing GS routinely in large-scale breeding programs. rAmpSeq is a simple, robust platform for designing primers, PCR amplification, and high-throughput multiplex sequencing, which allows hundreds to thousands of markers to be scored for less than $5 per sample (Buckler et al., 2016). rAmpSeq is specifically tailored to GS approaches (Voss-Fels et al., 2019). However, GS applications that include genotyping the training and validation sets with rAmpSeq markers have not been reported until now.

In the present study, an association-mapping panel and two maize DH populations, genotyped with GBS and rAmpSeq markers, were used to estimate the genomic prediction accuracy of kernel Zn concentration in maize. The main objectives were to: (1) estimate the genomic prediction accuracy for kernel Zn concentration in different maize populations using GBS and rAmpSeq markers; (2) compare the genomic prediction accuracies of kernel Zn concentration within and across multiple maize populations estimated by the different genotyping platforms; (3) assess the effect of training population size (TPS), marker density (MD) and marker quality on genomic prediction accuracy estimation; and (4) explore training population development base on the phenotypic variation of the target trait.

## Materials and Methods

### Plant Materials

An association-mapping panel and two DH populations were used to perform genomic prediction analyses in the current study. The association-mapping panel, designated Drought Tolerant Maize for Africa (DTMA), consists of 300 lowland tropical and mid-altitude tropical inbred lines. These lines originated from different CIMMYT maize breeding programs and have abundant genetic variation.

The two DH populations, designated DH1 and DH2, were derived from F1 crosses between two inbred lines. DH1 consisted of 108 lines, and the F1 cross was made between two elite maize inbred lines of CML503 and CLWN201. DH2 consisted of 143 lines, and the F1 cross was made between two elite maize inbred lines of CML465 and CML451.

### Field Trial and Zn Concentration Analysis

The DTMA panel was planted in Mexico at CIMMYT’s research stations in Agua Fria, Puebla, Mexico (20°27′N, 97°38′W; 110 m above sea level) during winter seasons (November–May) in the 2012–2013 and 2013–2014, and at the Instituto Nacional de Investigaciones Forestales, Agricolas y Pecuarias (INIFAP) station in Celaya, Guanajuato, Mexico (20°34′N, 100°49′W; 1,750 m above sea level) during summer season (May–November) in the 2012 (CE12B). The trials conducted during the winter season in 2012–2013 at Agua Fria and during the summer season at Celaya were laid out in a randomized complete block design with two replications and an alpha lattice design with two replications was used at Agua Fria in winter 2013–2014.

Two DH populations were planted at the INIFAP station in Celaya in the 2014 summer season (May–November), Tlaltizapan, Morelos, Mexico (18°41′N, 99°07′W, 940 m above sea level) in the 2015–2016 winter season (November–May), and Agua Fria in the 2017–2018 winter season (November–May). Single replication trials were planted in the Celaya and Tlaltizapan research stations. In the Agua Fria research station, the trials were planted using an alpha lattice design with two replications.

Plot size for all experiments was single row 2.5 m length, with 75 cm between rows, and 0.23 m between plants in each row. In each plot, six plants were self-pollinated, hand-harvested and hand-shelled to avoid any metal contamination, and bulked kernel samples from each plot were dried and sent for analysis at the maize nutritional quality analysis laboratory in Mexico. More details on the analyses of kernel Zn concentration have been previously described by Hindu et al. (2018).

### Phenotypic Data Analysis and Heritability Estimation

In each of the three populations, least-squares means of genotypes were calculated across environments through the “lsmeans” function of the R program, version 5.6.1 (R Core Team, 2019). The least-squares means were proposed to analyze the data with unequal subclass numbers, and which are predictions from linear or mixed models (Lenth, 2016). For the locations with replicated data, mean values across the two replications were calculated for predictions of least-squares means. A linear mixed model was fitted to the data as follow:

$$Y_{\text{ik}}=\mu +E\,n\,v_{\text{i}}+G\,e\,n_{\text{k}}+\varepsilon_{\text{ik}}$$

where *Y*_i__k_ is the mean performance of a certain genotype, μ is the overall mean effect, *Env*_*i*_ is the effect of *i*_th_ location, *Gen*_k_ is the main effect of the *k*_th_ genotype, *ε*_i__k_ is the error associated with the *i*_th_ location and the *k*_th_ genotype, which is assumed to be normally and independently distributed, with mean zero and homoscedastic variance. All factors except genotype were set as random.

Narrow-sense heritability was estimated as the ratio of additive genetic variance to total phenotypic variance:

$$h^{2}=\,V_{\text{A}}\,/\,V_{\text{P}},$$

where *V*_*A*_ was an estimate of the additive genetic variance, and *V*_P_ was the total phenotypic variance. The total phenotypic variance was the sum of *V*_A_ and *V*_e_, and *V*_e_ was an estimate of the residual variance. The variance components of *V*_A_ and *V*_e_ were estimated based on the genomic relationship matrix in the *rrBLUP* package (Endelman, 2011) of the R program, version 5.6.1 (R Core Team, 2019).

In both the DTMA panel and the two DH populations, the Pearson correlation coefficients of kernel Zn concentration between locations were estimated in each population using the R program version 5.6.1 as well.

### Genotyping and Genotypic Data Analysis

For all the lines in each population used in the present study, leaf samples of each line were collected 3–4 weeks after seeding for DNA extraction with a CTAB procedure (CIMMYT Laboratory protocol, 2005). All the lines in the two DH populations and a subset of the DTMA panel of 236 inbred lines were sent to the Biotechnology Resource Center of Cornell University for both GBS and rAmpSeq.

A GBS protocol commonly used by the maize research community was applied in this study (Elshire et al., 2011). Genomic DNA was digested with the restriction enzyme *ApeKI*, and a DNA library was constructed in 96-plex and sequenced on Illumina HiSeq2000. Details of analyses of SNP calling and imputation have been previously described (Cao et al., 2017). Initially, 955,690 SNPs evenly distributed on maize chromosomes were called for each line; 955,120 of them were assigned to chromosomes 1–10, and 570 of them could not be anchored to any of the 10 maize chromosomes.

rAmpSeq is a simple, robust, and cost-effective genotyping strategy developed by Cornell University for large-scale GS projects. Details of the rAmpSeq primer pair information have been described by Buckler et al. (2016). DNA libraries were constructed in 3072-plex and sequenced on Illumina HiSeq2000, and each sequence tag was treated as a unique dominant marker. Initially, 7,595 dominant markers identified from the intergenic regions were called for in each of the genotyped samples.

### Genomic Prediction Analysis Within Each Population

Genomic prediction was performed in the *rrBLUP* package in the R program version 5.6.1 (Endelman, 2011). The mixed model is described as:

y=X⁢β+Z⁢u+ε

where *y* is the vector (*n* × 1) of observations, *X* is the vector (*n* × 1) of individuals and β is the fixed effects, ε is the vector (*n* × 1) of independently random residuals with assumed distribution *N* (0, *I*σ_ε_^2^), *Z* is the design matrix (*n* × *m*) for random effects, and u is the vector of random effects with *u* ∼ *N* (0, *K*σ_u_^2^), *K* being an identity matrix in this case (Endelman, 2011; Liu et al., 2018). In addition, n is the number of individuals, and m is the number of markers.

A fivefold cross-validation scheme was used to generate the training and validation sets and assess the prediction accuracy within each population, where the average value of the correlations between the true breeding value and the genomic estimated breeding values was defined as genomic prediction accuracy. In each population, the data were divided into two subsets, with 80% of the lines randomly selected and assigned to the training set and the remaining 20% assigned to the validation set. In total, 150 replications of cross-validation were performed for each population. In the GBS dataset, TASSEL version 5.0 (Bradbury et al., 2007) was used to filter the markers in each of the three populations with a minor allele frequency (MAF) greater than 0.05 and a missing rate less than 20%. In total, 262,919, 65,430, and 46,426 GBS markers were selected for further genomic prediction analyses in the DTMA panel, DH1 population, and DH2 population, respectively. In the rAmpSeq dataset, markers with an MAF greater than 0.05 were filtered in each of the three populations. In total, 6,150, 3,859, and 2,795 markers were selected for further genomic prediction analyses in the DTMA panel, DH1 population, and DH2 population, respectively. In the same population, the prediction accuracies estimated from the GBS and rAmpSeq datasets were compared, and a *t*-test was conducted to obtain the significance.

### Effect of Training Population Size (TPS), Marker Density (MD) and Marker Quality on Genomic Prediction Accuracy Estimation

To assess the effect of TPS on the estimation of genomic prediction accuracy, the training population was set from 10 to 90%, with an interval of 10%, in each of the three populations. The number of markers used for prediction in each population were same as those used in the cross-validation analyses. In total, the analysis was repeated 100 times in each population.

To evaluate the effect of MD on the estimation of genomic prediction accuracy, the number of markers varying from 10 to all markers (i.e., 10, 50, 100, 300, 500, 1,000, 3,000, 5,000, 10,000, 50,000, and all markers) were used for genomic prediction analyses in each of the three populations. In the GBS datasets, all markers with an MAF greater than 0.05 and a missing rate less than 20% were filtered. In the rAmpSeq datasets, all markers with an MAF greater than 0.05 were filtered. The fivefold cross-validation scheme was repeated 100 times in each population with different marker datasets.

To examine the effect of marker quality on the estimation of genomic prediction accuracy, different levels of MAF and missing rates were used to filter the marker datasets and control marker quality. In GBS datasets, markers were filtered with the combinations between MAF and missing rate in each population; MAF ranging from 0.10 to 0.40, with an interval of 0.10; missing rates ranging from 0 to 80%, with an interval of 20%. In rAmpSeq datasets, markers were filtered with MAF in each population, and MAF ranging from 0.10 to 0.40, with an interval of 0.10. The fivefold cross-validation scheme was used to compare the prediction accuracies estimated from the marker datasets with different quality levels. In total, the analysis was repeated 150 times in each population.

### Training Set Development Based on the Phenotypic Variation of the Target Trait

Training sets were formed according to the phenotypic variation information of the target trait. Five scenarios were simulated and compared in each of the three populations, where the training set was formed by sampling the same percentage of genotypes with random selection (Random), with selection from the top tail (Top), with selection from the bottom tail (Bottom), with selection from the middle part (Middle), and with selection from the two tails (Two tails). In each scenario, the validation set was the whole population, and the training set ranged from 10 to 90%, with an interval of 20%. In each of the three populations, a total of 25 combinations and comparisons were conducted between the five scenarios and the five percentage levels of the training set.

### Genomic Prediction Analysis Between Pairwise Populations

Among the three populations, genomic prediction analyses between pairwise populations were performed, when one population was used as training set to predict the other population as validation set, the correlations between the true breeding value and the genomic estimated breeding values of the validation set was defined as genomic prediction accuracy. In the GBS dataset, TASSEL version 5.0 (Bradbury et al., 2007) was used to filter the markers across the three populations with a MAF greater than 0.05 and a missing rate less than 20%. In the rAmpSeq dataset, markers with an MAF greater than 0.05 were filtered across the three populations. The genomic prediction analyses between pairwise populations were performed with 137,593 and 6,005 markers selected from the datasets of GBS and rAmpSeq, respectively.

### Genomic Prediction Analysis With the Significantly Associated Markers

Genomic prediction analyses with significantly associated markers were performed to simulate marker assisted selection (MAS). In the previous study of Hindu et al. (2018), 20 GBS SNPs significantly associated with kernel Zn concentration in maize were identified in a collection of 923 inbred lines, and the DTMA panel used in the present study is a subset of these 923 inbred lines. In total, 11 of these 20 significantly associated SNPs were validated in three DH populations, and the two DH populations used in the present study are a subset of these three DH populations. A fivefold cross-validation scheme was used to assess the prediction accuracy of MAS within each population, when the 11 validated GBS SNPs were selected to perform genomic prediction. The comparison between GS and MAS was only applied in the GBS dataset, which is not applied in the rAmpSeq dataset, because of the lack of information about the physical position of the rAmpSeq markers.

## Results

### Phenotypic Variation

**FIGURE 1:**
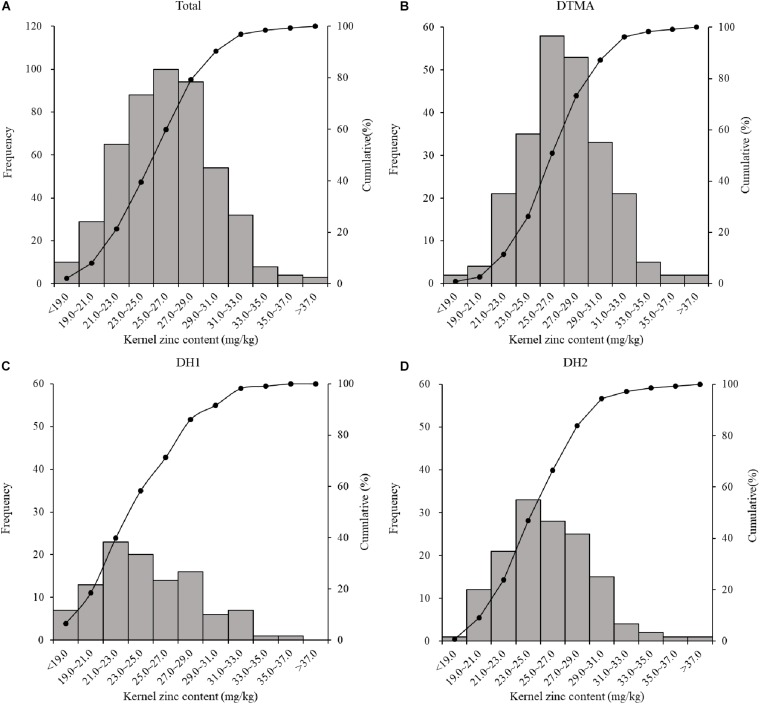
Phenotype distribution of maize kernel Zn conentration of: **(A)** all the inbred lines across the three population used in the present study; **(B)** all the inbred lines in the DTMA panel; **(C)** all the inbred lines in the DH1 population; **(D)** all the inbred lines in the DH2 population.

Kernel Zn concentration in the all genotypes across the three populations ranged from 16.87 to 39.53 mg/kg, with an average value of 26.11 mg/kg (Table 1). The average value of kernel Zn concentration was 27.11, 24.59, and 25.59 mg/kg in the DTMA panel, DH1 population, and DH2 population, respectively. The DTMA panel had the widest range of variation among the three populations, although the standard deviation within the two DH populations was similar to or higher than that within the DTMA panel. In the two DH populations, the greater standard deviation values indicated the larger genotype-by-environment interactions in a mixture of replicated and un-replicated trials, and the lower correlations between locations. In total, 2.87% of 487 inbred lines (i.e., 14 lines) reached 33.00 mg/kg (Figure 1), the target level of HarvestPlus, through bio-fortification (Bouis and Welch, 2010). Among the fourteen lines, nine were from the DTMA panel, four were from DH2, and only one was from DH1. The estimated narrow-sense heritabilities of the three populations were moderate to high, and the highest heritability (0.84) was observed in the DTMA panel, while the lowest heritability (0.62) was observed in DH2. In each population, the Pearson correlation coefficients of kernel Zn concentration between locations were moderate, ranging from 0.43 to 0.62 (details not shown).

**TABLE 1:** Basic information of three populations of DTMA association mapping panel, and two DH populations (DH1 and DH2), including population size, name of parents for DH populations, kernel Zn concentration in each population of the values of mean, minimum, maximum, and stand deviation, and heritability (*h*^2^), number of locations and number of replications.

**Population**	**Population size**	**Parent 1**	**Parent 2**	**Mean (mg/kg)**	**Minimum (mg/kg)**	**Maximum (mg/kg)**	**Stand deviation**	***h*^2^**	**Loc.**	**Rep.**
DTMA	236			27.11	18.35	39.53	3.41	0.84	3	6
DH1	108	CML503	CLWN201	24.59	16.87	36.45	4.01	0.75	3	4
DH2	143	CML465	CML451	25.59	18.38	37.93	3.50	0.62	3	4

### Distribution of MAF and Missing Rate

**FIGURE 2:**
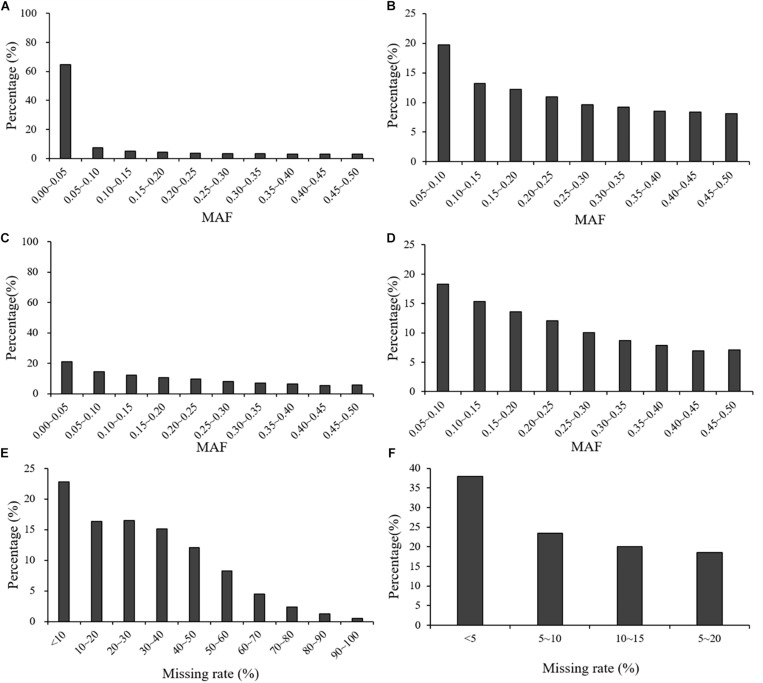
Distribution of MAF and missing rate across all the three populations before and after filtering: **(A)** MAF distribution of the GBS marker dataset before filtering; **(B)** MAF distribution of the GBS marker dataset after filtering; **(C)** MAF distribution of the rAmpSeq marker dataset before filtering; **(D)** MAF distribution of the rAmpSeq marker dataset after filtering; **(E)** missing rate distribution of the GBS marker dataset before filtering; **(F)** missing rate distribution of the GBS marker dataset after filtering.

In the GBS dataset across the three populations, the average MAF was 0.09, with continuous distribution classes from 0 to 0.50 at intervals of 0.05. Before filtering, 64.80% of the markers had an MAF < 0.05. In the other intervals (0.05–0.50), the percentages of markers in each interval were below 10%, ranging from 2.70 to 9.20%. After filtering, average MAF across three population had a significant increase to 0.23. In the GBS dataset across the three populations, the average missing rate across all the markers was 0.29 before filtering, and it decreased to 0.08 after filtering (Figure 2).

In the rAmpSeq dataset across the three populations, the average MAF was 0.19 and 35.40% of the total markers had an MAF < 0.05. In the other intervals (0.05–0.50), the percentages of markers in each interval ranged from 11.10 to 22.80% (Figure 2). After filtering, MAF across three population increased to 0.23.

### Genomic Prediction Accuracies Estimated From the Fivefold Cross-Validation Schemes Within Each Population

**FIGURE 3:**
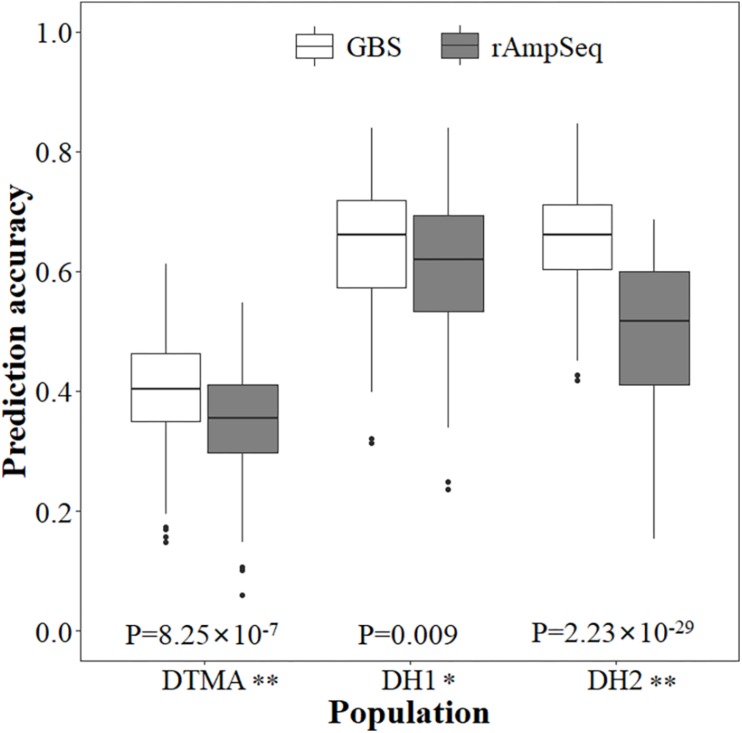
Genomic prediction accuracies of kernel Zn concentration in the DTMA panel, DH1 population, and DH2 population estimated with the GBS and rAmpSeq marker datasets.

Genomic prediction accuracies estimated from the fivefold cross-validation schemes for all three populations are shown in Figure 3, where the prediction accuracies were moderate and varied across populations and genotyping platforms. Among the three populations, the lowest prediction accuracy was observed in the DTMA panel across both the GBS and rAmpSeq marker datasets. In the same population, the prediction accuracy estimated from the GBS marker dataset was higher than that estimated from the rAmpSeq marker dataset, and the difference was significantly. The prediction accuracies estimated from the GBS marker dataset were 0.40, 0.64, and 0.65 for the DTMA panel, DH1 population, and DH2 population, respectively. The prediction accuracies estimated from the rAmpSeq marker dataset were 0.35, 0.61, and 0.50 for the DTMA panel, DH1 population, and DH2 population, respectively (Figure 3). In the same population, the prediction accuracies estimated from the GBS marker dataset had less variation than those estimated from the rAmpSeq marker dataset.

### Effect of TPS, MD, and Marker Quality on the Estimation of Genomic Prediction Accuracy

**FIGURE 4:**
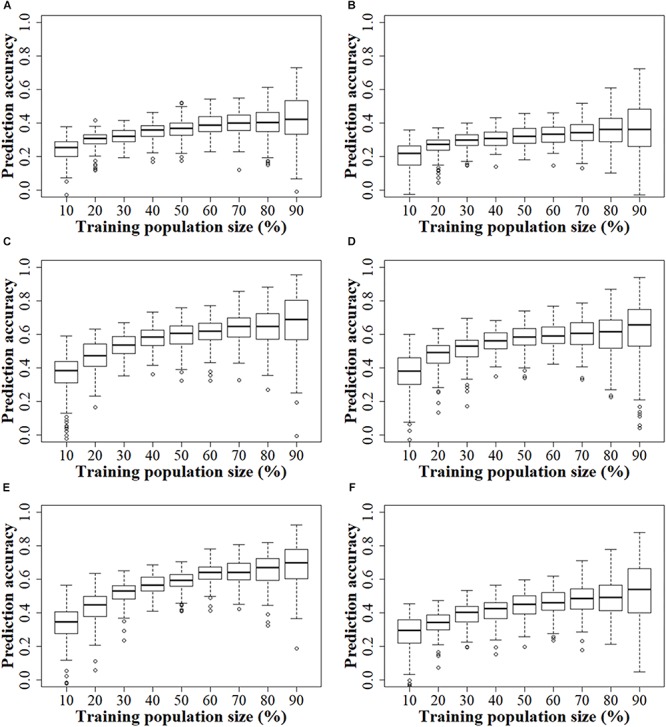
Genomic prediction accuracies of kernel Zn concentration in the DTMA panel, DH1 population, and DH2 population, when the training population size was set from 10 to 90% of total genotypes, with an interval of 10%. Panel **(A)** in the DTMA panel estimated with GBS markers; **(B)** in the DTMA panel estimated with rAmpSeq markers; **(C)** in the DH1 population estimated with GBS markers; **(D)** in the DH1 population estimated with rAmpSeq markers; **(E)** in the DH2 population estimated with GBS markers; **(F)** in the DH2 population estimated with rAmpSeq markers.

In both the GBS and rAmpSeq datasets, the prediction accuracy increased continuously as the TPS increased across all the populations (Figure 4). In the GBS datasets, the prediction accuracy increased slightly in both the DTMA panel and the two DH populations, when the TPS increased from 50 to 90%. The smallest standard error was observed in prediction accuracy when 60% of the total genotypes were assigned as the training set in the DTMA panel. In the two DH populations, the smallest standard error was observed when 50% of the total genotypes were assigned as the training set. In the rAmpSeq datasets, a similar trend was observed in all the three populations, which indicated that 50–60% of the total genotypes assigned as the training set can achieve good prediction accuracy.

**FIGURE 5:**
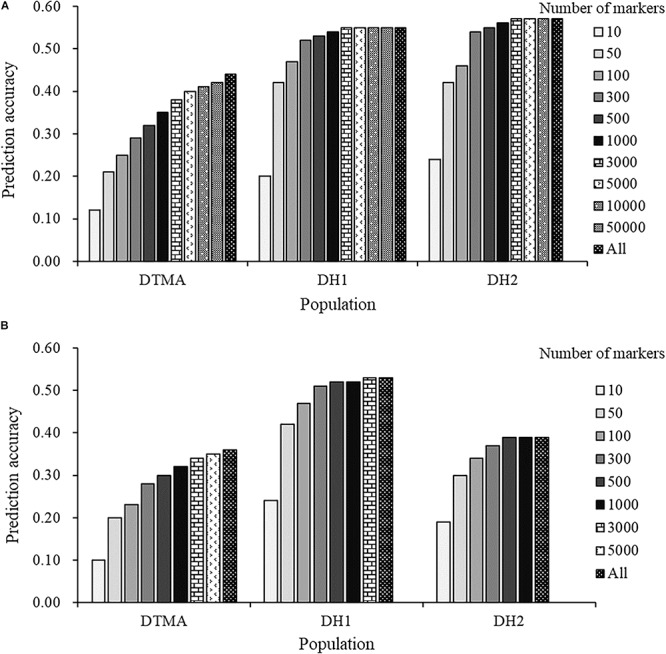
Genomic prediction accuracies of kernel Zn concentration in the DTMA panel, DH1 population, and DH2 population, with number of markers varying from 10 to all markers: **(A)** in the GBS marker dataset; **(B)** in the rAmpSeq dataset.

The effect of marker density on the estimation of prediction accuracy is presented in Figure 5. In the DTMA panel, the prediction accuracy continuously increased as the number of markers increased across both the GBS and rAmpSeq datasets. In the DH populations, a slight increase was observed in prediction accuracy when the number of markers increased from 300, the prediction accuracies nearly reached a plateau at 500 markers in both the GBS and rAmpSeq datasets. The results indicated that a larger number of markers is required to obtain higher prediction accuracy in populations with greater genetic diversity.

The result of prediction accuracies estimated in all the populations under the different marker datasets filtered with combinations of MAF and missing rate, or only with MAF, is presented in Tables 2, 3. This result showed a slight difference when compared with the prediction accuracies estimated with an MAF greater than 0.05 and a missing rate less than 20% in fivefold cross-validation schemes. In the GBS datasets across all the populations, MAF had a greater effect than the missing rate on the estimation of prediction accuracy, especially an MAF interval of 0.40–0.50 in the DH populations. When the MAF interval was 0.40–0.50, the average prediction accuracy estimated across all levels of missing rate was 0.40, 0.49, and 0.56 in the DTMA panel, DH1 population, and DH2 population, respectively, while the average prediction accuracy estimated across all missing rate levels in other MAF intervals ranging from 0.00 to 0.40, was 0.41, 0.65, and 0.66 in the DTMA panel, DH1 population, and DH2 population, respectively. Similar trends were observed in the rAmpSeq dataset across all the populations, a decreases in the prediction accuracy was shown in the MAF interval of 0.40–0.50.

**TABLE 2:** Genomic prediction accuracies of kernel Zn concentration in the DTMA panel, DH1 population, and DH2 population, estimated from the GBS marker datasets with different levels of quality filtered with missing rate and MAF.

**Missing rate**	**MAF**	**DTMA**		**DH1**		**DH2**	
		**Number of markers**	**Prediction accuracy**	**Number of markers**	**Prediction accuracy**	**Number of markers**	**Prediction accuracy**
0%	0.10	9656	0.39	14318	0.66	504	0.66
	0.20	5681	0.37	12294	0.65	495	0.66
	0.30	3314	0.35	6961	0.65	445	0.66
	0.40	1570	0.31	4330	0.44	329	0.51
20%	0.10	201258	0.39	64617	0.65	45440	0.66
	0.20	129155	0.42	55241	0.65	44080	0.65
	0.30	79223	0.42	31658	0.67	39934	0.65
	0.40	37999	0.41	19029	0.43	29699	0.57
40%	0.10	252221	0.43	94925	0.66	65411	0.66
	0.20	162792	0.43	80983	0.63	62977	0.66
	0.30	100366	0.43	47682	0.66	56738	0.67
	0.40	48312	0.42	26995	0.46	40970	0.58
60%	0.10	275811	0.42	120842	0.63	80595	0.65
	0.20	178326	0.42	103032	0.65	76640	0.66
	0.30	109965	0.43	62590	0.64	68022	0.65
	0.40	52972	0.43	35050	0.57	48562	0.58
80%	0.10	285127	0.43	137892	0.64	89870	0.67
	0.20	184501	0.42	116838	0.64	84845	0.65
	0.30	113833	0.41	71836	0.65	74818	0.65
	0.40	54893	0.41	39930	0.57	52811	0.56

**TABLE 3:** Genomic prediction accuracies of kernel Zn concentration in the DTMA panel, DH1 population, and DH2 population, estimated from the rAmpSeq marker datasets with different levels of quality filtered with MAF.

**Population**	**MAF**	**Number of markers**	**Prediction accuracy**
DTMA	0.10	4847	0.35
	0.20	2960	0.35
	0.30	1731	0.31
	0.40	811	0.27
DH1	0.10	3722	0.61
	0.20	3098	0.61
	0.30	1723	0.62
	0.40	1077	0.48
DH2	0.10	2588	0.5
	0.20	2392	0.49
	0.30	2096	0.52
	0.40	1429	0.45

Missing rate had a minor effect on the estimation of prediction accuracy, and a very slight difference in prediction accuracy was observed among the different levels of missing rate, except for the DTMA panel with a missing rate of 0%. Across all the MAF intervals, the average prediction accuracy estimated at the missing rate level of 0% was 0.36 in the DTMA panel, while the average prediction accuracy estimated in the DTMA across the other levels of missing rate was 0.42, with a range of 0.39 to 0.43.

### Training Set Development Based on the Phenotypic Variation of the Target Trait

**FIGURE 6:**
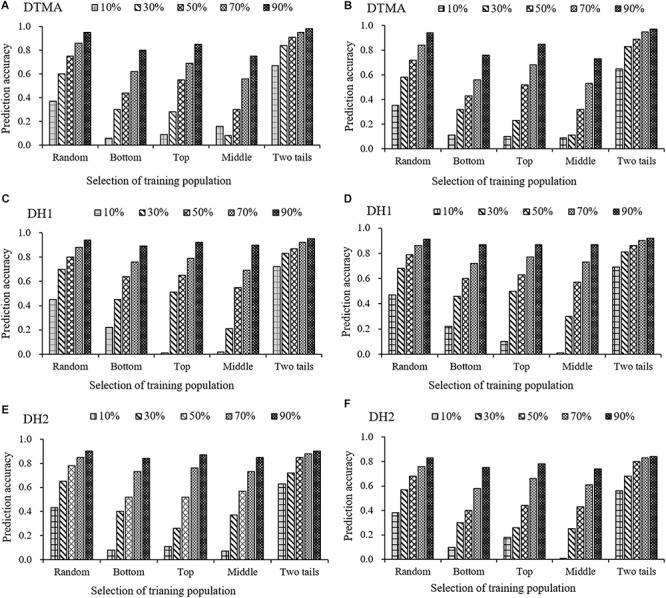
Genomic prediction accuracies of kernel Zn concentration in the DTMA panel, DH1 population, and DH2 population, when the training population was formed by sampling the same percentage of genotypes with random selection (Random), with selection from the bottom tail (Bottom), with selection from the top tail (Top), with selection from the middle part (Middle), and with selection from the two tails (Two tails). The training population ranged from 10 to 90% of the total genotypes, with an interval of 20%. Panel **(A)** in the DTMA panel estimated with GBS markers; **(B)** in the DTMA panel estimated with rAmpSeq markers; **(C)** in the DH1 population estimated with GBS markers; **(D)** in the DH1 population estimated with rAmpSeq markers; **(E)** in the DH2 population estimated with GBS markers; **(F)** in the DH2 population estimated with rAmpSeq markers.

For all three populations, the result of prediction accuracies estimated in the 25 combinations between the five scenarios and the five training set percentages are presented in Figure 6 for both the GBS and rAmpSeq datasets. Across all five scenarios and marker datasets, the prediction accuracy increased in all the populations as the increase of the percentages of training set. For example, the prediction accuracy estimated with the GBS dataset in the “Top” scenario was 0.04, 0.29, 0.58, 0.69, and 0.86, when the training set percentage in the DTMA panel was 10, 30, 50, 70, and 90%. Across all the training set percentages in all the populations, the “Two tails” scenario outperformed the other four scenarios in both the GBS and rAmpSeq datasets. For example, the prediction accuracy in the DTMA panel estimated with the GBS dataset at a training set percentage of 50% was 0.91, 0.75, 0.55, 0.30, and 0.44 for the Two tails, Random, Top, Middle, and Bottom scenarios, respectively. Similar trends were also observed in the two DH populations. These results indicated that developing a training set with broad phenotypic variation is possible to improve the prediction accuracy.

### Genomic Prediction Accuracies Estimated From the Pairwise Populations

Genomic prediction accuracies between pairwise populations are shown in Table 4, where the prediction accuracies were very low across all the pairwise populations and genotyping platforms. In the GBS dataset, the average prediction accuracy across the six pairwise populations was 0.04. The highest prediction accuracy value was 0.32, when the DTMA panel was used as training set to predict the DH1 population as validation set. The prediction accuracies in other five pairwise populations were close to zero. In the rAmpSeq dataset, the average prediction accuracy across the six pairwise populations was 0.08. The highest prediction accuracy value was 0.24, when the DH2 population was used as training set to predict the DH1 population as validation set. The second highest prediction accuracy value was 0.19, when the DTMA panel was used as training set to predict the DH1 population as validation set.

**TABLE 4:** Genomic prediction accuracies between pairwise populations estimated from the GBS and rAmpSeq marker datasets.

	**Training set**	**Validation set**	**Prediction accuracy**	
			**GBS**	**rAmpSeq**
	DTMA	DH1	0.30	0.19
		DH2	−0.12	−0.13
	DH1	DTMA	0.05	0.07
		DH2	0.05	0.15
	DH2	DTMA	−0.06	−0.04
		DH1	−0.02	0.24

### Prediction Accuracy of MAS Estimated With the Significantly Associated SNPs

Genomic prediction accuracies estimated with the significantly associated SNPs are shown in Table 5, where GS outperformed MAS and showed higher genomic prediction accuracies within each of the three populations. The average prediction accuracy of MAS in the populations of DTMA, DH1, and DH2 was 0.22, 0.49, and 0.42, respectively. The average prediction accuracy of GS in the populations of DTMA, DH1, and DH2 was 0.40, 0.64, and 0.65, respectively (Table 5 and Figure 3).

**TABLE 5:** Comparison the prediction accuracy between GS and MAS estimated from the fivefold cross-validation scheme within each of the three populations, the prediction accuracies of GS were estimate from the filtered GBS dataset, and the prediction accuracies of MAS were estimate from the significantly associated SNPs.

**Population**	**GS**				**MAS**			
	**Maximum**	**Minimum**	**Mean**	**Standard deviation**	**Maximum**	**Minimum**	**Mean**	**Standard deviation**
DTMA	0.61	0.15	0.40	0.09	0.53	−0.16	0.22	0.12
DH1	0.88	0.31	0.64	0.12	0.82	−0.02	0.49	0.16
DH2	0.85	0.42	0.65	0.09	0.76	0.01	0.42	0.14

## Discussion

The main advantage of GS over phenotype-based selection is that it can accelerate the genetic gain per unit time and unit cost by reducing the selection cycle time and the phenotyping cost. However, the prediction accuracy must be high enough for GS to be effective. In the present study, an association-mapping panel and two maize DH populations genotyped with GBS and rAmpSeq markers were used to estimate the genomic prediction accuracy of kernel Zn concentration in maize. Results indicated that the prediction accuracies of kernel Zn concentration in maize were moderate to high and varied across populations and genotyping platforms. The prediction accuracy of kernel Zn concentration in the association panel estimated with GBS and rAmpSeq markers was 0.40 and 0.34, respectively. In the two DH populations, the prediction accuracies of kernel Zn concentration estimated with GBS markers ranged from 0.64 to 0.65, while the prediction accuracies of kernel Zn concentration estimated with rAmpSeq markers ranged from 0.50 to 0.61.

In the same population, the prediction accuracy estimated from the GBS marker dataset was higher than that estimated from the rAmpSeq marker dataset, and the difference was significantly. Several genomic prediction studies using GBS markers were implemented successfully in maize to improve various traits with different levels of genetic complexity, where the genotyping cost was at least $35 per sample at the 96-plex level, or $13 per sample at the 384-plex level (Wu et al., 2016). However, a high-throughput genotyping platform with affordable genotyping cost is still required for implementing GS routinely in the large-scale breeding programs. A low-cost genotyping platform allows GS to be more cost-effective, and makes it feasible to replace expensive phenotyping with cheaper genotyping. The total breeding population size increases under the same budget by phenotyping a lower number of breeding lines in the training set and genotyping a greater number of breeding lines in the prediction set. Therefore, the selection intensity increases to accelerate the genetic gain per unit cost. rAmpSeq is specifically tailored to GS approaches, with a cost of $5 per sample. To the best of our knowledge, this is the first report of a genomic prediction study in maize using rAmpSeq markers, and the results of this study showed that GS using rAmpSeq markers is a cost-effective approach to improve the kernel Zn concentration in maize through bio-fortification. GS using rAmpSeq markers is also being implemented in CIMMYT maize breeding programs for improving grain yield and kernel Zn concentration simultaneously. The preliminary cost-benefit analysis showed that a breeding strategy that implements phenotype-based selection and GS stepwise could reduce the breeding cost up to 50% compared with phenotype-based selection only, equivalent to double the total breeding population size under the same budget.

Results of this study showed that the prediction accuracies continuously increased as the TPS increased in all the populations. Across the two genotyping platforms, relatively high prediction accuracies with the smallest standard error were observed in all populations, when 50 to 60% of the total genotypes were used as a training set. Results of this study are consistent with previous reports (Crossa et al., 2014; Cao et al., 2017), where the results also recommend phenotyping and genotyping as few as half of the genotypes as the training set to achieve good prediction accuracy of the target trait. In addition, prediction accuracy could be further improved by polling multiple populations as the training set to increase the population size, which will be assessed in further studies.

The transferability of the GS models across populations was assessed by estimating the prediction accuracies between pairwise populations, when one population was used as training set to predict the other population as validation set. The prediction accuracies were very low across all the pairwise populations and genotyping platforms, the prediction accuracies in a few pairwise populations are above or close to 0.20, which indicates that the genomic prediction accuracies estimated across populations could be improved furtherly by increasing the TPS and strengthening the relationship between the training and prediction sets. The transferability of the GS models across populations and across years are being tested with a larger dataset from a maize breeding program, where the TPS is bigger and the relationship between the training and prediction sets is closer. The preliminary results are promising, and more details will be reported in further studies.

How marker density affects prediction accuracy has been investigated in several previous studies, and the number of markers required to achieve good prediction accuracy may vary depending on the extent of linkage disequilibrium between markers and QTL, population types, and the genetic complexity of the target trait. In this study, the results showed that the prediction accuracy of kernel Zn concentration in maize continuously increased as the number of markers increased across populations and genotyping platforms. Across the two genotyping platforms, the prediction accuracy reached a plateau at 3,000 markers in the DTMA panel, and at 500 markers in the two DH populations, which indicated that a larger number of markers are required to obtain higher genomic prediction in populations with greater genetic diversity. In the tropical maize association-mapping panel, the average linkage disequilibrium decay distance over all 10 chromosomes was less than 5 kb at *r*^2^ = 0.1, and roughly an estimated 500,000 markers are required to ensure that at least one marker can be in linkage disequilibrium with trait-associated loci. The DH population has a clear genetic structure and finite chromosome recombination events (Smith et al., 2008). Therefore, less than 500 markers are enough to ensure that at least one marker can be in linkage with each gene-related locus.

There is a tradeoff between number of markers and marker quality, because marker quality becomes lower as the number of markers increase in a specific marker dataset. The number of markers affecting prediction accuracy has been investigated in several previous studies, but very few reports have been conducted to estimate the effect of marker quality on prediction accuracy estimation. Results of this study show that the prediction accuracy reached a plateau at 3,000 markers in the DTMA panel, and at 500 markers in the two DH populations. It indicated that more markers with lower quality have little effect on prediction accuracy improvement, and markers with lower quality could be the noise on improving prediction accuracy. Moreover, the computational burden increases when a higher number of markers are used for prediction. Appropriate levels of MAF and missing rate should be considered and selected to improve the prediction accuracy and reduce the computational burden by balancing the number of markers and marker quality.

Several previous studies estimated the genetic diversity of the training set with molecular markers, and assessed the genetic diversity of the training set on prediction accuracy improvement (Dos Santos et al., 2016; Norman et al., 2018). Results of this study indicated that the genetic diversity of the training set could be estimated not only with molecular markers, but also with phenotypic data. Therefore, prediction accuracy can be improved by developing a training set with broad phenotypic variation.

In the present study, GS outperformed MAS and showed higher genomic prediction accuracies within each population on predicting the kernel Zn concentration. These results showed that kernel Zn concentration in maize could be improved by implementing GS individually or by implementing MAS and GS in a stepwise fashion. As we discussed earlier, a breeding strategy that implements phenotype-based selection and GS stepwise could reduce the breeding cost up to 50% compared with phenotype-based selection only, equivalent to double the total breeding population size under the same budget, if the breeding target is to improve grain yield and kernel Zn concentration simultaneously. The long-term genetic gain of how much GS could provide to improve grain yield and increase the Zn concentration will be further assessed in CIMMYT maize breeding programs. Alternative, kernel Zn concentration in maize could be improved by implementing MAS and GS in a stepwise fashion, where MAS is implemented as forward breeding at an early generation on larger numbers of selection candidates, followed by GS at advanced stages of breeding on smaller number of selection candidates for further improvement. The decision of a breeding strategy to implement MAS and GS stepwise for improving kernel Zn concentration in maize requires further research.

## Data Availability Statement

The datasets generated in this study can be found in the following repository: https://data.cimmyt.org/dataset.xhtml?persistentId=hdl:11529/10548362.

## Author Contributions

XZ, LZ, and TD initiated and designed the overall study. NP-R, FS, and TD performed and coordinated the field experiments and phenotypic data collection. XZ, MO, ML, and BP contributed to the genotypic data generation. RG, EM, DY, YR, AZ, and JC carried out the statistical analysis. RG, XZ, LZ, and TD interpreted the results and wrote the manuscript. All authors contributed to manuscript editing.

## Conflict of Interest

The authors declare that the research was conducted in the absence of any commercial or financial relationships that could be construed as a potential conflict of interest.

## Footnotes

1 www.harvestplus.org
